# Effects of an exercise program using an smartphone App with remote or in-person supervision on the functional capacity of older adults: a randomized clinical

**DOI:** 10.31744/einstein_journal/2026AO1757

**Published:** 2026-03-30

**Authors:** André Issao Kunitake, João Carlos Ferrari Corrêa, Raphael Mendes Ritti-Dias, Fernanda Ishida Corrêa

**Affiliations:** 1 Doctoral and Master's Program in Rehabilitation Sciences Universidade Nove de Julho São Paulo SP Brazil Doctoral and Master's Program in Rehabilitation Sciences, Universidade Nove de Julho, São Paulo, SP, Brazil.

**Keywords:** Frail older individuals, Functional residual capacity, Postural balance, Muscle strength, Mobile applications, Physical exercise

## Abstract

**Introduction:**

Population aging is a significant global challenge, demanding strategies that promote healthy aging. Regular physical activity is crucial for maintaining functional capacity, quality of life, and preventing chronic diseases in older adults. However, sedentary behavior remains high, and evidence on effective, supervised smartphone-based exercise strategies for older adults is limited.

**Objective:**

To evaluate the effects of smartphone-based exercises with in-person or remote therapeutic supervision on functional capacity.

**Methods:**

Thirty-four older adults were randomized into two training groups with App-based exercises: a Control Group with in-person supervision and an Experimental Group with remote supervision. The exercises were performed three times a week for 8 weeks. The Experimental Group performed exercises at homes with remote supervision, and the Control Group performed exercises in the laboratory. Functional capacity was measured using the Glittre Activities of Daily Living (Glittre ADL) test, postural balance with the Mini Balance Evaluation Systems Test, and lower limb strength with the Five Times Sit-to-Stand test.

**Results:**

Both groups demonstrated significant improvement in functional capacity. After 8 weeks, Glittre-ADL test time reduced similarly in both groups (Experimental Group: 217±63 to 188±47 s and Control Group 194±34 to 171±30 s, ES=0.09). Postural balance improved similarly in both groups (ES=0.15), and the lower limb strength improved in the Experimental Group (ES=0.32).

**Conclusion:**

A smartphone App-based exercise program, with and without in-person supervision, improved functional capacity and postural balance in older adults, whereas lower limb strength improved only with in-person supervision. Brazilian Registry of Clinical Trials: RBR-22ctkjq.

## HIGHLIGHTS

Smartphone-based training improved functional capacity in older adults.

Remote and in-person supervision led to similar gains in functional capacity.

Postural balance improved comparably in both groups.

Lower limb strength improved only in-person supervision with exercise App.

## In Brief

This study evaluated App-based exercise programs with in-person or remote supervision in older adults. After 8 weeks, both groups showed similar improvements in functional capacity and postural balance. Lower limb strength improved only in the in-person supervised group, supporting the effectiveness of smartphonebased training.

## Graphical abstract

**Figure:**
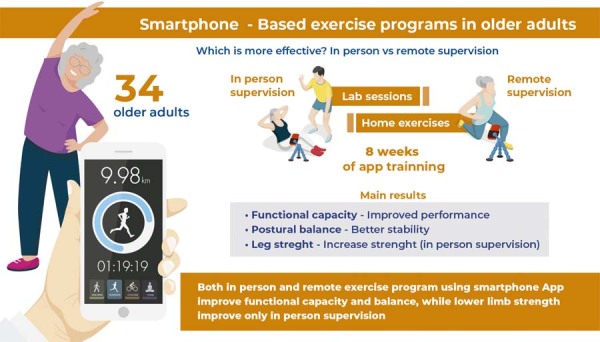

## INTRODUCTION

Global population aging is the most relevant medical and social demographic problem worldwide. UK Office for National Statistics (2012)^( 1 )^ predict a 23% increase in the population over 60 years of age by 2035.

Therefore, strategies promoting healthy aging are required. The World Health Organization^( 2 )^ defines healthy aging as maintaining social inclusion and immune to diseases or disabilities that impair daily activities and reduce functional capacity.

Thus, regular physical activity is crucial for the health of older adults, promoting physical and mental well-being, improving functional capacity and quality of life, and helping to prevent or reduce chronic disease (hypertension, diabetes) progression.^( 3 - 6 )^

Despite these well-described and known effects, a systematic review^( 7 )^ showed that older adults remain sedentary for an average of 9.4 h a day, and Taylor (2014)^( 8 )^ notes that many do not comply with current physical activity recommendations. This low adherence stems from barriers such as limited access to sports and leisure activities and lack of transportation.^( 9 , 10 )^

Thus, smartphones have been used to encourage healthy habits such as physical exercise because they can be adapted to different environments and carried out at any time of the day, improving adherence by practitioners.^( 11 , 12 )^ Furthermore, smartphone- and tablet-guided exercise is a highly feasible, well-accepted, reliable, and easily implemented, allowing older adults exercise with greater autonomy.^( 12 - 14 )^ In addition, smartphone use among older adults is increasing.

Smartphone applications (Apps) may be more cost-effective than traditional interventions and enable a pragmatic approach to decentralized, equitable healthcare.^( 15 - 17 )^ However, a major challenge is that many older adults find it difficult to exercise alone because they need guidance and proper instructions to maximize benefits and prevent risks such as falls, muscle injury, and fractures.^( 18 , 19 )^

## OBJECTIVE

This study aimed to evaluate the effects of exercises using a smartphone application with in-person or remote therapeutic supervision, on functional capacity and motivation in older adults.

## METHODS

### Trial design

This study was a randomized controlled clinical trial involving older adults. Participants were assigned either to training using a remotely supervised exercise App (Experimental Group - EG) or to training using an exercise App under face-to-face supervision (Control Group - CG). Functional capacity (primary outcome), postural balance, lower-limb strength, satisfaction, motivation, and attendance (secondary outcomes) were obtained at baseline, after 4 weeks, and after 8 weeks of intervention.

### Participants

Fifty older adults residing in São Paulo, Brazil, were recruited. Inclusion criteria required participants to be between 60 and 80 years old (with an upper age limit to ensure sample homogeneity), physically active, and functionally independent with a Katz^( 20 )^ score ≥4. Participants also had to be vaccinated against coronavirus disease (COVID-19), exhibit preserved cognitive function (assessed using the Mini-Mental State Examination,^( 21 )^) and have no neurological, cardiovascular, or musculoskeletal diseases contraindicating physical exercise. All participants provided written informed consent.

All assessments were conducted in the Rehabilitation Sciences laboratory of the Master’s and Doctoral programs at *Universidade Nove de Julho* , São Paulo, Brazil. Participants in the Experimental Group performed the exercises in the laboratory, and Control Group participants performed the exercises at home.

### Interventions

Participants were randomized to the Control Group (CG), which performed the App-based exercises in the laboratory during individual sessions supervised by a therapist, or to the Experimental Group (EG), which completed App-based exercises at home. The EG could contact the therapist via phone or message for clarification or guidance if they had difficulties performing the exercises. Furthermore, therapists frequently contacted patients to identify any complications.

The exercise program was conducted using the free application ‘Exercise for Older Adults’ available for Android and iOS platforms. All patients were familiar with smartphones. The researcher installed the application on the participants’ smartphones and instructed them to use it before performing the activities.

The training program used the ‘Improve your strength and balance’ exercise category, which included warm-up exercises, stationary running, free movement and small jumping tasks, and lower limb strength exercises (such as sit-to-stand, squats and lunges) abdominal exercises and bi- and single-leg balance exercises; Table 1S , Supplementary Material). The EG received a booklet explaining how to perform each exercise, based on the application manual.

**Table 1S:** Exercise Booklet

Instructions:
1) You are receiving a booklet with guidelines for exercises that will be performed using the application.
2) The exercise program should conducted 3 days a week.
3) Before performing exercises, measure your blood pressure with the pressure measuring device and ensure you are feeling comfortable, without shortness of breath or anxiety.
4) Make sure you are wearing comfortable clothing, not too tight:
T-shirt (tank top, cotton, nylon);
Pants (sweatshirt, nylon pants, or leggings; avoid jeans);
Footwear (sneakers or closed shoes that do not slip)
5) Prepare the exercise area
Do not leave many objects nearby, except for the chair and supports;
Use a firm, heavy chair to prevent it from tipping over or moving out of place during exercises.
6) Exercises
Abdominal exercises are performed on a mat. If you don’t have one, it can be done lying down in your bed;
The exercises are performed standing close to a wall.
Walking exercises should preferably be performed in a hallway or near a wall to support yourself if you lose your balance.
All exercises are timed and must be performed with the application installed on your smartphone/cell phone.
Always rest after each exercise.
**Run in place** Perform movements as if you were running but without leaving the place (do it for 1 minute)
**Boxing movements** Perform movements as if you are throwing punches (perform for 1 minute).
**Walk by raising your leg high, bending your knee and hip (perform for 1 minute)**
**Single limb stance** Stand behind a chair or fixed surface, support yourself with one hand. Lift your right foot off the floor and try to balance on your left leg. Try to maintain this position for as long as the exercise lasts. Repeat the same exercise on the other side. The objective of this exercise is to try to stand on one foot without holding onto the chair, maintaining this position for as long as possible.
**Walking heel to toe** You will walk in a straight line positioning your right foot in front of your left foot, such that your right heel touches the tip of your left foot’s toes. Move your left foot in front of your right, touching your heel to the toes of your right foot. Repeat this movement during the exercise time.
**Chair squat** Stand with your feet hip-width apart and in front of a chair. Keeping your chest straight and looking straight ahead as you slowly lean your butt back, bending your knees and slowly sitting up. Touch your butt to the chair or sit on it. Tilt your torso slightly forward to stand up. Stand up slowly until reaching an upright posture. Repeat this movement for as long as the exercise lasts. Be careful not to fall during the movements.
**Lunges** Stand with your feet hip-width apart. Contract your abdomen. Take a big step forward with your right leg. Slowly start to squat, trying to bring your knee close to the floor. Be careful not to hit your knee hard on the floor. Always keep your torso straight. Avoid placing the knee of the right leg too far in front of the right foot. Return to the starting position and repeat the same movement on the other side.
**Rock the boat** Stand with your feet hip-width apart. Make sure both feet are firmly placed on the floor. Keep your head up looking straight ahead. Shift your body weight onto your right foot and slowly lift your left leg off the floor. Try to maintain this position for as long as the exercise lasts. Repeat the same movement on the other side, slowly lifting the opposite leg.
**Clock reach** You will need a chair for this exercise. Imagine that you are in the center of a clock. Number 12 is right in front of you and number 6 is right behind you. Hold the chair with your left hand. Raise your right leg and stretch your right arm pointing forward. Point your arm to the side, then behind you. Bring the arm back to the side, then forward. Keep your head straight and looking forward at all times. Repeat this exercise on both sides.
**Bicycle crunches** Lie on the floor or bed, face up. Place your hands behind your head and try to pedal while keeping your abdomen contracted throughout the movement. As you bend your knee, try to bring your opposite elbow toward it, until it touches your knee. Ensure your rib cage is moving (rotating) with your elbow movements.
**Back leg raise** Stand behind a chair. Slowly lift your right leg back without bending your knees or stretching your toes. Hold this position for a second, then gently lower your leg. Repeat the movements alternating feet with each left and right leg.
**Single limb stance with arms** Stand with your feet together and arms at your sides next to the chair. Raise your left hand over your head and slowly lift your left foot off the floor. Hold this position for 10 seconds. Repeat the same action on the right side.
**Side leg rise** You will need a chair for this exercise. Stand behind the chair with your feet slightly apart. Slowly lift your right leg out to the side. Keep your back straight, your big toe pointed forward and look straight ahead. Lower your right leg slowly. Repeat this movement alternating feet.

Participants were required to perform the exercises three times a week for 8 consecutive weeks, totaling 24 sessions. Each session lasted approximately 45 min, with duration varying according to the rest interval between each exercise. Participants selected their preferred rest interval time, which could vary between 30 and 60 s.

As a protective measure, the therapist assessed the CG participants’ blood pressure and heart rate before and after exercise using an automatic sphygmomanometer.

The EG was instructed to have these measurements taken before and after each exercise session, subsequently reporting the values to the researcher.

### Outcomes

To characterize the sample, demographic data collected including age, weight, height, and body mass index (BMI) was collected using a questionnaire. Symptoms of depression and sleep-related disorders were assessed using the Beck Depression Inventory^( 22 )^ and the Pittsburgh Sleep Quality Index,^( 23 )^ respectively, to characterize the sample.

Primary and secondary outcomes were measured pre- and post-intervention

### Primary outcome - functional capacity

Functional capacity was assessed using the Glittre Activities of Daily Living (ADL) test, previously validated for older adults by Gomes et al.^( 24 )^ The test began when participants rose from a chair (without upper limb support, 46cm from feet to seat), while wearing a backpack (2.5kg for women or 5.0kg for men). Participants subsequently walked 10m on a flat track and halfway up and down two steps (step measurements 17cm high ×27cm deep) and continued walking towards a shelf where there were three objects weighing 1kg. Each weight was transferred, one at a time, with both hands, from the upper shelf (at the height of the participant’s shoulders) to the lower shelf (at the level of the participant’s pelvic girdle) and from there to the floor. Thereafter, participants placed the objects again on the central shelf and then on the top shelf, returned along the 10-meter route, went up and down the steps, and sat back on the chair ( Figure 1 ).

**Figure 1:**
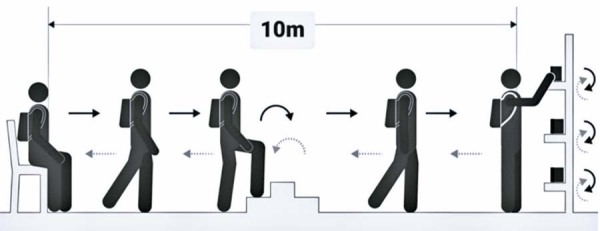
Glittre Activities of Daily Living (ADL) test

Own Source

This circuit was repeated five times, as quickly as possible, without running. The shorter the time (s) to perform the test, the better the individual’s functional capacity. The predicted time (s) of the Glittre-ADL test was calculated for adult participants based on age, BMI, and height as independent variables, as described by Reis et al.,^( 25 )^ using the Glittre test formula: predicted ADL=0.558 + (0.018 × BMI) + (0.016 × age years).

### Secondary outcomes

Postural balance was measured using the Mini-Balance Evaluation System Test,^( 26 )^ assessing balance during anticipatory postural adjustments, postural responses, sensory orientation, and gait. The score varied from 0 to 28 points; the higher the score, the better the balance.

Lower limb strength was evaluated with the Five Times Sit-to-Stand test.^( 27 )^ This measures the time it takes the individual (s) to rise from a chair with a backrest and no armrests to a standing position, repeating the movement five times.

### Level of satisfaction and motivation with the exercise program

Participant satisfaction and motivation with the exercise program were assessed using a researcher-developed questionnaire. The questionnaire contained the following questions: Were you satisfied with the exercise App? (Yes or no, and then rate from zero to 10). Were you satisfied with the exercise program? (Yes or no, and then rate from zero to 10). How motivated are you with the exercise program? (Not motivated, slightly motivated, moderately motivated, very motivated, and extremely motivated).

### Sample size

The sample size was based on a pilot study with ten participants, five participants per group. Using an effect size of 0.55, an α=0.05, and β=0.8, the estimated required sample size was 28 participants. However, owing to dropout risk, an additional 20% of the sample was included, resulting in a total sample of 34 participants.

### Randomization and blinding

An independent researcher randomized the participants into the EG and CG using block randomization via www.randomizer.org. A blinded researcher, unaware of each participant’s exercise program allocation, conducted assessments.

### Statistical methods

Data normality was assessed using the Shapiro-Wilk test. Continuous data were expressed as the mean±standard deviation, and categorical data as absolute and relative frequencies. Baseline group comparisons were performed using the Independent t-test and Fisher’s exact test. The effects of exercise training programs were analyzed using the Generalized Estimation Equation. Eta squared (η^2^ ) was presented as a measure of effect size. For inferential analysis, statistical significance was set at p<0.05.

## RESULTS

Fifty older adults were contacted between January 2022 and July 2022, and sixteen were excluded from the study for the following reasons: aged >80 years (n=4), fear of the COVID-19 pandemic (n=8), and lack of interested in participating (n=4) ( Figure 2 ).

**Figure 2:**
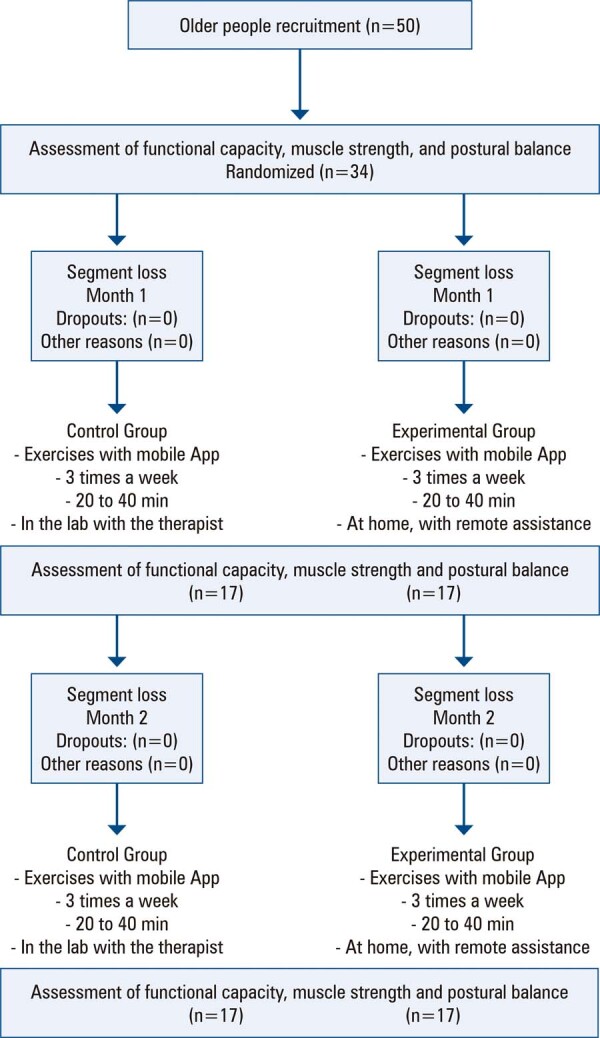
Study flowchart. Application (App), laboratory (lab)

The final sample included 34 participants, and their characteristics are presented in table 1 .

**Table 1:** Baseline demographic and clinical characteristics for Control and Experimental Groups

Variable	Control Group (n=17)	Experimental Group (n=17)	p value
Clinical profile			
Gender, % female	88	94	1.00
Age, yrs.	71 (6)	68 (4)	0.09
Weight, kg	64 (12)	70 (13)	0.13
Height, m	1.59 (0.05)	1.59 (0.15)	0.87
Body mass index, kg/m^2^	25.4 (5.2)	27.9 (5.2)	0.18
Systolic blood pressure, mmHg	124 (16)	118 (8)	0.24
Diastolic blood pressure, mmHg	81 (11)	78 (4)	0.41
MMSE, score	27 (3)	26 (4)	0.31
Katz Index, score	5.9 (0.3)	5.9 (0.2)	0.56
BDI, score	10 (9)	8 (6)	0.49
PSQI, score	6 (2)	7 (4)	0.36
Outcomes at baseline			
Glittre- ADL test, sec	217 (63)	194 (34)	0.19
Predicted Glittre - ADL test, s	190 (5)	187 (4)	0.47
Mini BEST test, score	23 (4)	25 (2)	0.06
Five times Sit-to-Stand test, s	10 (3)	10 (2)	0.90

Data presented as the mean±standard deviation or relative frequency.

BDI: Beck Depression Inventory; PSQI: Pittsburgh Sleep Quality Index; MMSE: Mini Mental State Examination.

At baseline, the groups were similar for all clinical variables and outcomes (p>0.05).

**Figure 3:**
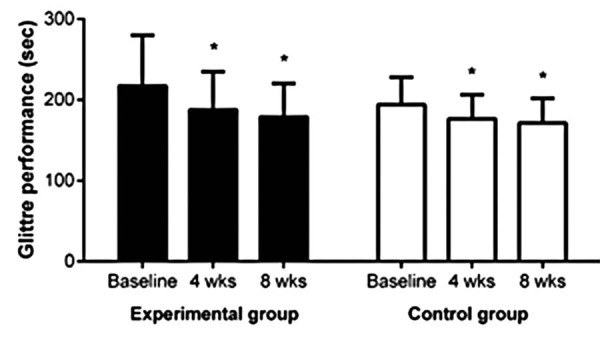
Glittre-ADL test performance in seconds at baseline, after 4 weeks (wks), and 8 weeks of intervention for the Experimental and Control Groups

All participants completed interventions without complications. Pre- and post-session blood pressure values consistently remained within the acceptable range throughout the exercise program in both groups (Figure 1S, Supplementary Material). Figure 3 presents functional capacity results of both groups at baseline, 4 weeks, and 8 weeks after the intervention.

* Significant difference from baseline p<0.05.

Data are expressed as the mean ± standar derivation

Both groups showed significant, comparable improvement in Glittre-ADL test performance from baseline to 4 and 8 weeks (EG: 217±63 s to 188±47 s after 4 weeks, and 179±41 s after 8 weeks; CG: 194±34 s to 177±30 s after 4 weeks, and 171±30 s after 8 weeks; time effect p<0.01, ES=0.09).

Individual analysis (Figure 2S - Supplementary Material) showed that all older participants increased their functional capacity after 8 weeks of training.

Table 2 presents the Mini Balance Evaluation System Test and the Five Times Sit-to-Stand test results. Both groups showed significant, comparable Mini Balance Evaluation System Test improvements from baseline to 4 and 8 weeks (time effect p<0.01). Five Times Sit-to-Stand performance improved at 8 weeks only in the CG (group x time effect, p<0.05).

**Table 2:** Mini Balance Evaluation System Test (Mini-BEST) and five times sit-to-stand results

	Control Group			Experimental Group			Effect size Baseline to 4 weeks	Effect size Baseline to 8 weeks
	Baseline	4 weeks	8 weeks	Baseline	4 weeks	8 weeks		
Mini-BEST Score (0-28)	23.4 (3.6) (15-27)	26.8 (1.4)* (23-28)	27.2 (1)* (24-28)	25.4 (1.9) (22-28)	26.8 (1.1)* (24-28)	27.2 (0.9)* (25-28)	0.18	0.15
Five times Sit-to-Stand (s)	10.1 (2.7) (7-17)	10.1 (3.3) (6-19)	8.2 (2.2)* (6-15)	10.0 (1.8) (7-13)	9.7 (1.6) (7-13)	9.7 (2.0) (6-13)	0.01	0.32

Data expressed, mean, standard deviation, minimum, and maximal.

* Significant difference from baseline p<0.05.

Table 3 reports attendance, motivation, and satisfaction outcomes with exercise programs for both groups. Attendance, motivation, and satisfaction were similar between groups (p>0.05).

**Table 3:** Attendance, motivation, and satisfaction with exercise

	Control Group (n=17)	Experimental Group (n=17)	p value
Attendance, sessions (0-24)	23.1±1.7 (18-24)	22.2±2.3 (18-24)	0.19
Motivation, score (1-5)	4.9±0.3 (4-5)	4.6±0.4 (4-5)	0.11
Satisfaction, score (1-10)	9.8±0.4 (9-10)	9.6±0.5 (9-10)	0.14

Data presented as the mean±standard deviation and minimum and maximal score.

## DISCUSSION

This study showed that an exercise program using a smartphone application improved functional capacity and balance in older adults with and without in-person supervision. However, lower limb strength improved only when exercises were accompanied with in-person supervision. The smartphone-based exercise program was considered motivating and satisfactory regardless of supervision modality.

The primary novelty of this study is the finding of a smartphone application-based exercise program improved older adults’ functional capacity regardless of the supervision strategy. These results align with a prior study^( 27 )^ of adults aged 18-50, which showed than an application enhanced functional movement, flexibility, strength, and cardiovascular conditioning, indicating its potential improve physical performance. Similarly, Lyons et al.^( 28 )^ demonstrated that an intervention combining physical activity wearable monitoring a tablet and telephone counseling was feasible and acceptable among adults aged 55-79.

The improvement in participants’ functional capacity in this study was clinically relevant. At baseline, both the Control and Experimental Groups performed the ADL Glittre test performance was slower than expected for this age.^( 25 )^ After 2 months of training, the Control Group reduced test time by 38 s and the Experimental Group by 23 s. Consequently, the ADL Glittre performance after the training programs was faster than the age-predicted average for both groups.

Participants’ postural balance improved after the exercise program regardless of supervision strategy, aligning with Papi et al.,^( 29 )^ who observed improved anteroposterior (A/P) and mediolateral (M/L) orthostatic posture oscillation in older adults undergoing a balance exercise program. Silveira et al.^( 12 )^ also observed that assistive technology using tablets helped and motivated healthy older independent adults to perform strength and balance exercises independently with low dropout rates. Finally, Delbaere et al.^( 30 )^ showed that an App-based at-home balance exercise program did not significantly reduce fall frequency yet lowered fall rates and fall-related injuries over 2 years, indicating promise as a fall prevention strategy for community-dwelling older adults.

Lower-body strength gains revealed a significant group × time interaction, with improvements observed only in the in-person supervised group. This suggested that direct supervision-related factors, such as increased safety, precise exercise prescription, and immediate feedback, may enhance the intervention’s effectiveness.^( 31 , 32 )^ Such support may be particularly important for lower-limb strength exercises directly related to sit-and-stand test performance, such as sit-to-stand movements, squats, and lunges, where concerns about falls and adequate exercise intensity may arise.

In-person programs are the preferred option among older adults^( 33 )^ and individuals with chronic conditions such as Parkinson’s disease^( 34 )^ and cancer.^( 35 )^ In contrast, the present findings indicated no differences in attendance, motivation, and satisfaction between groups, suggesting that remotely delivered programs may be as effective as in-person interventions. This equivalence may be partly attributable to the convenience of remote formats, eliminating travel demands – a notable barrier in large urban centers such as São Paulo. Moreover, participants perceived improvements in physical function, including maintenance or enhancement of overall physical activity, aerobic capacity, balance, and general functional capacity, likely reinforced adherence, motivation, and satisfaction. Contextual factors, such as COVID-19-related restrictions may also have strengthened intrinsic motivation and contributed to the absence of dropouts. Finally, App usability also appeared to facilitate engagement, as commonly reported barriers to technology use among older adults were not observed in this study. Collectively, these elements may explain the positive perception of the remote intervention comparable to in-person programs.

This study design included a short-term, 8-week intervention, leaving the medium- and long-term stability of the findings uncertain. Remote programs can maintain adherence for up to 6 months^( 36 )^ or longer, although use/willingness usually decline in over time. Retention is not always complete yet feasible. Interventions with additional support, such as feedback or peer support, demonstrate greater retention, highlighting the importance of motivational and social mechanisms.^( 37 , 38 )^ Future studies should evaluate whether the results observed in the current study persist over extended periods.

Although adherence was comparable between groups, a potential limitation of this study is that the App used for the remote intervention provided instructions only in English, necessitating adaptation and translation into the participants’ native language to ensure proper comprehension of the exercises. Despite written instructions, some participants in the in-person supervised group still required direct guidance to perform activities correctly, indicating that language barriers constrained autonomy for certain participants. Consequently, enhanced outcomes might have been attained if the App had provided instructions in the participants’ native language.

Another limitation is the narrow age range of participants (60-80 years), selected to increase sample homogeneity and ensure feasibility and safety of the clinical trial given that the intervention requirements for smartphone use, active monitoring, and a certain level of digital literacy. Consequently, although benefits were demonstrated for this age group, the findings cannot be directly generalized to the oldest-old or institutionalized populations without further investigation.

Future research should consider adaptive designs or pilot studies specifically targeting this population, incorporating objective reality, measures preparatory technology use training, enhanced safety monitoring, and age group-stratified analyses to evaluate intervention effectiveness and safety in more vulnerable subgroups.

## CONCLUSION

In conclusion, this study indicated that a smartphone application-based exercise program improved functional capacity and postural balance and was considered motivating and satisfactory for older adults when performed with and without in-person supervision. However, improvements in lower limb strength occurred only with in-person supervision.

The findings highlight the potential of smartphone applications as a strategy to enhance health outcomes in older adults. These results broaden the scope for prescribing and monitoring exercise programs among older adults, including for individuals unable or unwilling to receive supervised interventions. Further investigation over an extended duration is warranted to ascertain whether the observed levels of participant motivation, exercise frequency, and physical improvements persist over time.

## Supplementary Material

**Figure 1S:**
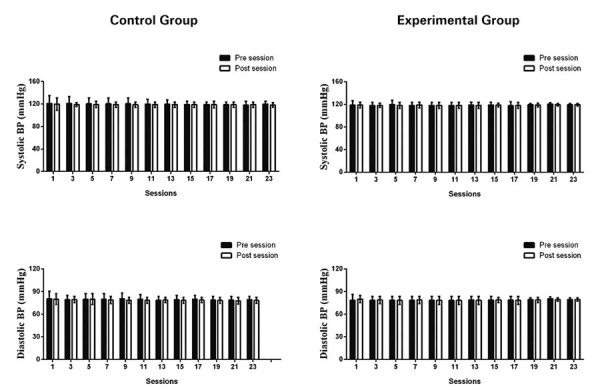
Systolic and diastolic blood pressure responses before and after each intervention throughout the intervention program in Experimental and Control Groups

**Figure 2S:**
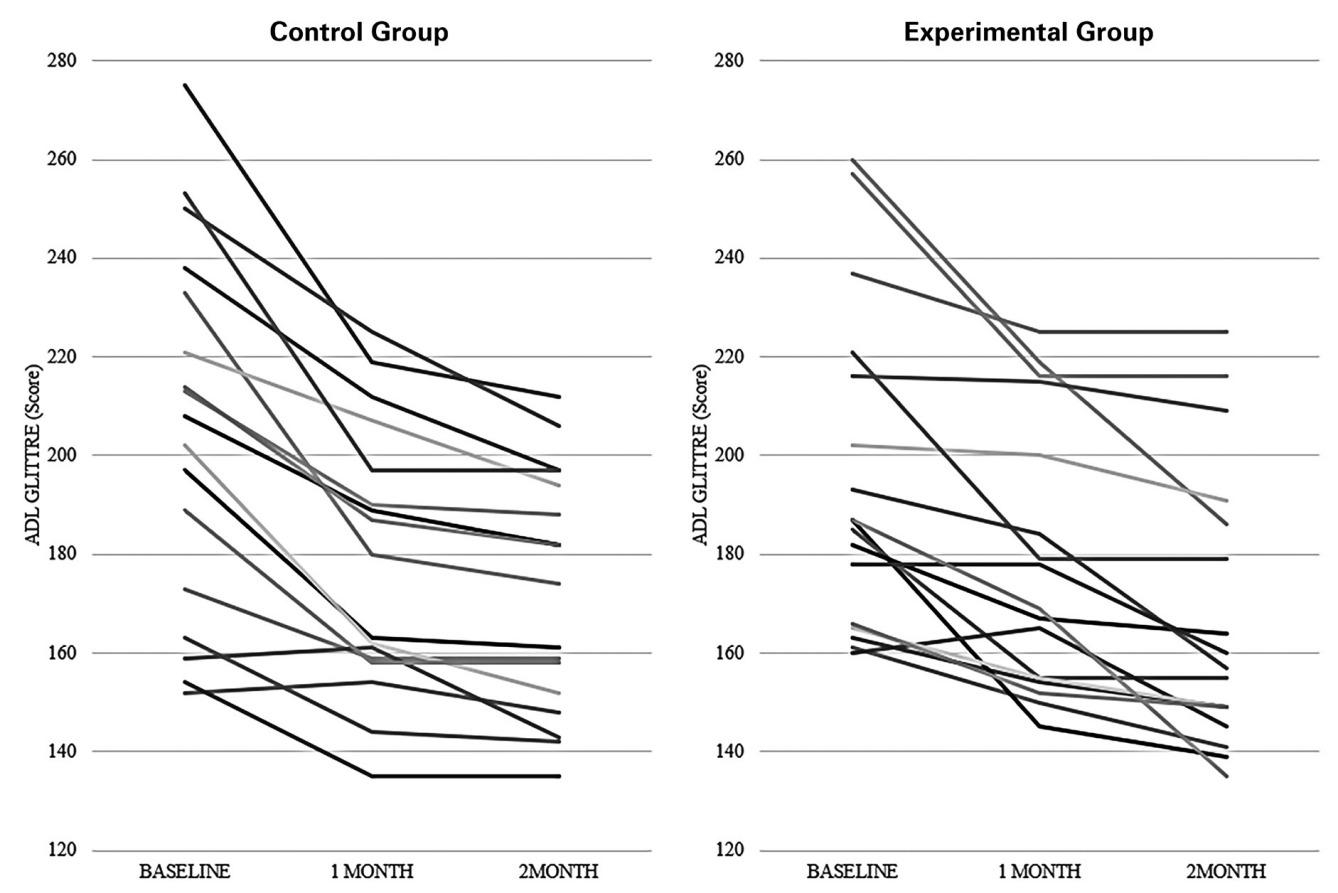
Line chart of Control and Experimental Groups at baseline, after 1 month, and after 2 months of training
